# Recent Advancements in Research on DNA Methylation and Testicular Germ Cell Tumors: Unveiling the Intricate Relationship

**DOI:** 10.3390/biomedicines12051041

**Published:** 2024-05-08

**Authors:** Alina-Teodora Nicu, Ileana Paula Ionel, Ileana Stoica, Liliana Burlibasa, Viorel Jinga

**Affiliations:** 1 Genetics Department, Faculty of Biology, University of Bucharest, 030018 Bucharest, Romania; nicu.alina-teodora@s.bio.unibuc.ro (A.-T.N.); ileana.stoica@bio.unibuc.ro (I.S.); 2 Department of Specific Disciplines, Faculty of Midwifery and Nursing, University of Medicine and Pharmacy “Carol Davila”, 050474 Bucharest, Romania; 3 Department of Urology, Faculty of Medicine, University of Medicine and Pharmacy “Carol Davila”, 050474 Bucharest, Romania; viorel.jinga@umfcd.ro; 4 The Academy of Romanian Scientists, 050044 Bucharest, Romania

**Keywords:** testicular germ cell tumors, DNA methylation, liquid biopsy, biomarkers, epidrugs, DNMTi, infertility

## Abstract

Testicular germ cell tumors (TGCTs) are the most common type of testicular cancer, with a particularly high incidence in the 15–45-year age category. Although highly treatable, resistance to therapy sometimes occurs, with devastating consequences for the patients. Additionally, the young age at diagnosis and the treatment itself pose a great threat to patients’ fertility. Despite extensive research concerning genetic and environmental risk factors, little is known about TGCT etiology. However, epigenetics has recently come into the spotlight as a major factor in TGCT initiation, progression, and even resistance to treatment. As such, recent studies have been focusing on epigenetic mechanisms, which have revealed their potential in the development of novel, non-invasive biomarkers. As the most studied epigenetic mechanism, DNA methylation was the first revelation in this particular field, and it continues to be a main target of investigations as research into its association with TGCT has contributed to a better understanding of this type of cancer and constantly reveals novel aspects that can be exploited through clinical applications. In addition to biomarker development, DNA methylation holds potential for developing novel treatments based on DNA methyltransferase inhibitors (DNMTis) and may even be of interest for fertility management in cancer survivors. This manuscript is structured as a literature review, which comprehensively explores the pivotal role of DNA methylation in the pathogenesis, progression, and treatment resistance of TGCTs.

## 1. Introduction

Testicular cancer has a germ cell histology in approximately 90–95% of cases, which fall under the category of testicular germ cell tumors (TGCTs). This represents the most prevalent tumor type among men in the 15 to 45 age bracket and is a significant cause of mortality within this age group [1,2]. Considering the young age at which they are diagnosed, there is a great concern for patients’ fertility because of the testicular origin and localization, as well as the toxicity associated with anticancer treatments.

According to the latest WHO classification, TGCTs are categorized based on their origin into germ cell neoplasia in situ (GCNIS)-related and non-GCNIS-related categories, with the latter being further subdivided into pediatric teratomas/yolk sac tumors and spermatocytic tumors [3]. They are also denoted as type II (GCNIS-related), type I, and type III, respectively. Type II tumors are subdivided into seminomas (SGCT) and nonseminomas (NSGT), with the latter comprising embryonal carcinomas (ECs), yolk sac tumors (YSTs), teratomas (TEs), and choriocarcinomas (CCs). Although type I and type II share histology, their origin, development, and molecular characteristics are distinct. In prepubertal tumors, teratomas are benign and further transition to malignant yolk sac tumors, while in type II tumors, teratomas are malignant and originate from embryonal carcinomas, which can also differentiate into yolk sac tumors [4,5]. Thus, it is important to note the category when referring to these subtypes. Type III tumors have a spermatogonial or spermatocytic origin and are quite rare. We will further refer to type II tumors simply as TGCTs, unless otherwise noted, as previous studies have mostly adopted this denomination. Prior to it, the changes in classification made the interpretation of results more difficult due to mixing of subtypes in studies, and the high number of mixed tumors is additionally problematic. For convenience, a partial classification including only the most prevalent types can be found in Table 1. The rarest types and variants are omitted as they can be diagnosed as rarely as once in a decade, and research on these types is extremely limited.

TGCTs have a high survival rate, yet NSGCTs tend to be more aggressive and can easily spread in the early stages [1,5]. A particular concern is raised by the resistance to current treatment options, which include surgery, radiation therapy, and chemotherapy. Chemotherapy involves the use of cisplatin, bleomycin, and etoposide, sometimes in a combination known as BEP therapy [6]. Cisplatin is a genotoxin long used for the treatment of TGCT, as well as other types of cancer, with good efficiency [7]. However, resistance to chemotherapy occurs in up to 10–15% of cases and leads to death in the absence of alternative treatment [8,9]. As such, novel therapeutic approaches are under investigations, including immunotherapy, poly ADP-ribose polymerase inhibitors (PARPis), and epigenetic drugs. The mechanisms behind chemoresistance are also being investigated, particularly the involvement of epigenetic modifications in cisplatin resistance [10].

Epigenetic modifications hold particular significance in TGCTs, besides the possible therapeutic connections. There seems to be a major involvement of epigenetic mechanisms in the initiation and progression of cancer, which has become evident in recent years as major efforts have been made to identify risk factors and potential biomarkers [11]. Considering that no major genetic factors seem to be strong candidates, and different hormonal and environmental risk factors are associated with epigenetic modifications, the interest has switched towards studying the epigenome of TGCTs [11,12,13]. A growing body of work now supports the importance of epigenetic studies related to TGCTs, covering DNA methylation, histone modifications, chromatin remodeling, and RNA interference [13].

**Figure 1:**
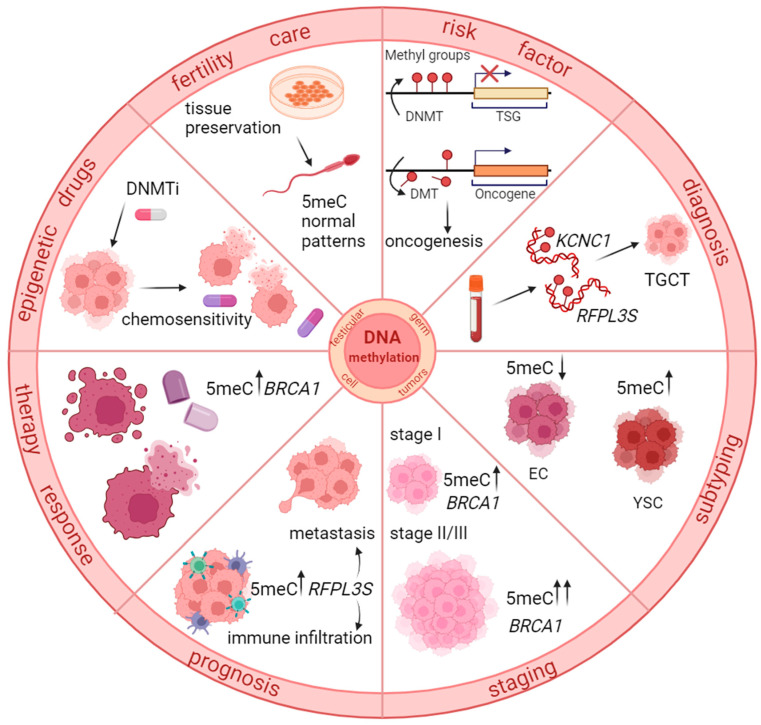
A schematic representation of the main directions of study for TGCT patients’ improved care, with DNA methylation at its core. (Legends: 5meC—5-Methylcytosine; BRCA1—breast cancer 1 gene; RFPL3S—ret finger protein-like 3S; DNMTi—DNA methyltransferase inhibitor; DMT—demethylases; KCNC1—potassium voltage-gated channel subfamily C member 1; TSG—tumor suppressor gene; EC—Embryonal carcinoma; YSC—Yolk sac tumor; ↑—High level; ↓—Low level).

DNA methylation is the most studied epigenetic mechanism as it plays an essential role in germline development. Thus, it was the first epigenetic mechanism investigated in association with TGCT and continues to be widely studied. Over the years, it has become increasingly evident that DNA methylation is involved in all major aspects of this cancer type. Therefore, our literature review provides a new approach for studying the recent associations between DNA methylation and TGCT, integrating results from the main directions of research, namely risk factors, biomarker identification, and therapeutic and infertility implications, as summarized in Figure 1.

The studies included in this review were selected based on their relevance to the role of DNA methylation in testicular germ cell tumors. The literature search was confined to publications between the years 1980 and 2024; however, given the existent body of work, we chose to focus on papers published during the past 5 years in order to present the most recent advancements in the field.

## 2. DNA Methylation and Risk Factors for Cancerogenesis

The main mechanism involved in normal male germline development, which could have implications for TGCT development, is represented by epigenetic reprogramming, a process in which DNA methylation plays a central role. The alterations that occur during epigenetic reprogramming bear consequences on the fertilization capacity of spermatozoa or even on the offspring, despite further reprogramming that takes place in the embryo [14,15]. Germline epigenome alterations are also thought to be involved in the emergence of the precursor lesion GCNIS [14].

DNA methylation usually refers to the addition of a methyl group to the 5′ cytosine of the dinucleotide CpG, although there are alternative variants, such as the methylation of adenine or of different sites (e.g., CHH or CHG, where H stands for adenine, thymine, or cytosine) [16]. This addition is catalyzed by DNA methyltransferase enzymes (DNMTs), using S-adenosyl-L-methionine as a substrate [17]. Of these enzymes, DNMT1 is responsible for maintaining DNA methylation patterns when cells divide, DNMT3A and DNMT3B are responsible for de novo methylation, and DNMT3L has no methyltransferase activity but mediates DNA methylation by interacting with DNMT3A/B [18,19]. DNA methylation facilitates gene inactivation, transposable elements silencing, X chromosome inactivation and, as mentioned, epigenetic reprogramming. Therefore, DNMT enzymes are essential for proper development, and their expression is highly regulated.

Epigenetic reprogramming of the embryo entails quick global demethylation under the action of demethylases (DMTs), which ensures totipotency and de novo methylation during the blastocyst stage. DNA methylation is maintained in imprinted genes and transposable elements as their silencing is essential for development. The reprogramming of the male germline begins in the sixth week of pregnancy when primordial germ cells (PGCs) migrate to the genital ridge. At this time, methylation patterns are erased, but they will later be reestablished in a sex-specific manner so that paternal imprinting, as well as transposable elements silencing, occur [14,20]. PGCs, together with somatic cells, form gonads, where spermatogenesis will take place. Spermatogenesis involves mitotic divisions of PGCs into spermatogonia, which later divide, generating additional spermatogonia for stock replenishing and primary spermatocytes that will divide meiotically. Through meiotic divisions, primary spermatocytes generate secondary spermatocytes and consequently spermatids, which will undergo maturation into spermatozoa [20]. When it comes to methylation patterns, spermatogonia will have stem cell patterns, while spermatozoa will be highly methylated. DNMTs expression is decreased as progression from spermatogonia to consequent cell types occurs [21]. Besides DNA methylation, all epigenetic mechanisms are involved in ensuring the proper development of the embryo and germline [15].

Improper DNA methylation patterns are involved in cancer initiation, mainly by two mechanisms: hypomethylation of oncogenes, which leads to their activation, and hypermethylation of tumor suppressor genes, which leads to their repression. Global alterations, however, are frequently observed. GCNIS cells exhibit a demethylated genome. Thus, it is believed that these lesions originate during embryonic development. Similarly, seminomas show global hypomethylation, whereas non-seminoma subtypes exhibit varied methylation patterns, contingent on their level of differentiation. Two distinct subtypes of seminomas seem to exist according to their bearing of mutations in the *KIT* gene, in which the wild type seminomas have a higher level of methylation [4]. Another look at seminoma subtypes has revealed one subtype with a higher pluripotency state and demethylated genome, while the second subtype shows a higher level of methylation with attributes of non-seminomas [22]. Overall, seminomas more strongly resemble PGCs and express pluripotency genes, particularly *OCT3/4*, *SOX2*, and *NANOG* [23,24].

Embryonal carcinomas have shown similarities with pluripotent stem cells in terms of their methylation levels of CpG and non-CpG sites [25]. Accordingly, they have come to be regarded as the malignant equivalent of embryonic stem cells [26]. This subtype is thought to differentiate to TE and YST, adopting a somatic-resembling methylation state that includes the methylation of pluripotency genes [25]. Similar to seminomas, non-seminomas express OCT3/4 and NANOG, with the addition of SOX17 [24].

Given the absence of identifiable genetic risk factors and the supposed epigenetic involvement, there have been attempts to identify epigenetic risk factors instead. The current body of work is limited to few differentially methylated genes. *RASSF1A* hypermethylation has been proposed as a risk factor in a couple of studies, followed by a meta-analysis with results confirmed in a cohort of 32 patients [27,28,29]. *RASSF1A* promoter hypermethylation was identifiable in the peripheral blood of TGCT patients, both in the seminoma and non-seminoma groups. Although stratification by tumor subtype was not possible because of the limited number of patients, the meta-analysis provided additional evidence. In the same study, *MGMT* was not differentially methylated between TGCT patients and the control group, although a couple of studies associated promoter hypermethylation of *MGMT* with TGCT [30,31]. Due to the difficulty of establishing strong risk factors, a switch has been made towards diagnostic and prognostic biomarkers instead, as further discussed.

## 3. DNA Methylation as a Biomarker

DNA methylation is proposed as a promising non-invasive biomarker for various cancers, including TGCT. These biomarkers, detectable in bodily fluids like blood, urine, and sperm, lay the foundation for liquid biopsy techniques. Of these fluids, blood represents a convenient source, as cell-free DNA hypermethylation can be detected in serum or plasma; although, sensitivity is reduced when looking at a single gene, and the use of a panel would provide better results. Additionally, several technical challenges have been noted, such as higher levels of cell-free DNA correlated with leukocyte contamination in samples that are not immediately processed, or even in serum over plasma [32]. Because circulating DNA originating from tumors actually has a low burden in plasma and serum samples, some studies have even proposed using whole blood for biomarker detection, acknowledging the fact that the additional presence of blood cells may provide valuable information as their genetic and epigenetic profiles can be altered in cancer and tumor circulating cells and are also a significant source of abnormal DNA [33,34,35].

Although liquid biopsies represent a much more convenient alternative to conventional practices, especially for early diagnosis, their use has been hindered in TGCT by more than just technical challenges [36,37]. Obtaining data related to TGCT is particularly difficult as it has a low incidence; consequently, very few studies have been conducted on patients. Furthermore, the number of patients is limited in these few studies. Additionally, considering the diverse subtypes, it can be troublesome to achieve the statistical significance that would give the study a high relevance. Various cell lines have been generated as well as even animal models, ranging from xenografted to genetically engineered cases for certain subtypes [38,39,40,41]. However, research advancement has been slow compared with more common cancer types (e.g., prostate, breast, etc.) because of the small number of reproducible results. The past few years have seen a boom in -*omics* and high-throughput studies, which has led to the development of invaluable databases that can be exploited through bioinformatics for quick discoveries in silico, which can then be confirmed in vitro and/or in vivo. Such is the case of The Cancer Genome Atlas (TCGA), a public database that provides extensive information on 33 cancer types generated from over 20,000 samples, both tumor and normal [42]. In addition to the deep molecular characterization of cancers, numerous tools are available for data processing and visualization. The usage of TCGA has now become common, and several pan-cancer studies have been conducted in the past couple of years [43,44,45]. However, normal tissue information is not available for all types of cancer, which constitutes a limitation in some studies that include TGCTs. This limitation can be overcome by the additional use of other public databases, like the Genotype-Tissue Expression (GTEx) database, which contains normal testis expression data that enable the necessary comparisons [46]. Still, the number of TGCT patients is relatively low, even in the TCGA database, with a current number of 263 cases, of which 150 cases provide DNA methylation data [42]. What is more, different TGCT subtypes have poor representation as most of them are seminomas, mixed tumors, and embryonal carcinomas (*n* = 150, *n* = 55, and *n* = 27, respectively), which leaves only one choriocarcinoma, three teratomas, and four yolk sac tumors (type II GCT), with the addition of two teratocarcinomas and five benign teratomas (type I GCT) [42]. Thus, expanding the dataset is essential for more informative results.

Nevertheless, with the help of datasets such as those available from TCGA, many recent studies take a pan-cancer approach, investigating risk factors, potential biomarkers, or therapeutic targets across a multitude of cancer types. So far, multiple studies have included TGCT data but have obtained underwhelming results. For instance, *FANCD2*-upregulated expression may be involved in tumor initiation, development, and progress and shows significant prognostic value in various types of cancers, including hepatocellular carcinoma, lung adenocarcinoma, and non-muscle invasive bladder cancer [47]. A decrease in methylation correlated with its overexpression was observed in a pan-cancer TCGA-based study which included TGCT, but no significant results were obtained for this type [47]. Another example concerns Cytoplasmic FMR Interacting Protein 2 (*CYFIP2*), which is a p53-inducible gene possibly involved in tumor initiation and progression. A recent pan-cancer study included TGCT in their investigation of *CYFIP2* but found no significant alterations [48]. Other pan-cancer studies found either upregulated or downregulated genes, but no significant changes in their methylation levels. The first category includes genes such as *DLD*, *PSAT1*, and *APOE*, while the second category includes genes such as *ACAP1* and *LIAS* [49,50,51,52,53].

A family of important tyrosine kinase receptors, TRK, coded by *NTRK* genes, is already a therapeutic target, with two inhibitors approved in patients with gene fusion mutations. A pan-cancer analysis examining the *NTRK* expression level and methylation status found mRNA-high mutations in TGCT patients, involving copy number variations (CNVs). These mutations occurred in *NTRK3*, whose methylation status seemed to be altered as well, showing a significant downregulation; however, no prognostic value was deemed relevant [54].

While some of these results may seem disappointing, they provide valuable information for researchers, who can focus their resources on other directions, building on previous work. Alternatively, future research may elucidate some of the unclear or incomplete findings. For instance, *SERPINH1* was found to be overexpressed in TGCT and was strongly correlated with immune infiltration [55]. The authors suggested that it may influence tumor progression in multiple cancers through MMR-mediated DNA repair and DNA methylation due to a strong association with DNMTs expression, including in TGCT [55]. Additional investigations may examine its methylation levels and its possible use as a prognostic biomarker.

Another pan-cancer TCGA-based study found that colony-stimulating factor 3 receptor (CSF3R) is up-regulated in TGCT, and its promoter’s methylation level was found to be lower in non-seminomas compared with seminomas [56]. Moreso, its expression was positively correlated with the tumor infiltration of cancer-associated fibroblasts and dendritic cells. CSF3R was found to be associated with cancer-associated pathways, and further studies could determine whether *CSF3R* methylation values can be used as a prognostic marker [56]. A closely related gene, *CSF1R*, had previously shown prognostic value by association with poor outcome when overexpressed through hypomethylation [57]. Additional genes whose upregulation was associated with hypomethylation are *FCER1G*, *SLC2A1*, and *AKT3* [58,59,60]. These genes showed different prognostic values, indicating tumor proliferation (e.g., FCER1G), immune infiltration, metastases, or poor overall survival, as summarized in Table 2. Some genes, such as *BMP1*, were upregulated and showed strong prognostic value for overall survival, progression-free survival, or tumor immune infiltration, but had a positive correlation with DNA methylation; thus, they did not correspond with the usual observations of gene hypomethylation–gene overexpression [61].

Another pan-cancer study showed that limb-bud and heart (LBH) was overexpressed in TCGT, and its levels were associated with poor prognosis [67]. *LBH* codes for a tissue-specific transcription cofactor, being a direct target of the oncogenic WNT/ß-catenin pathway. Further analyses show that hypomethylation of *LBH* is correlated with its overexpression. Additionally, LBH high levels were found to be associated with WNT hyperactivation, but unfortunately, those further observations did not include TGCT data [67].

WNT signaling is of particular interest as it regulates major cellular processes involved in differentiation, proliferation, migration, and apoptosis, which has led to its identification as a major player in cancer when dysregulated. The gain of WNT activators or loss of WNT repressors leads to aberrant WNT signaling, which is why Xu and al. looked at mutations and methylation levels in a cohort of children and adolescents with germ cell tumors (type I GCT), as well as adult data (type II) from TCGA [68]. Their study found low levels of promoter methylation in WNT pathway activators and high levels in WNT inhibitors, both in type I and type II GCT. Moreover, they showed prognostic significance for WNT activation, which was associated with poor survival. Several WNT inhibitors are under development as therapeutic agents for restoring proper pathway functioning. Two of them showed promising results for GCT in vitro in four cell lines and in vivo in a zebra fish model [68]. Other cellular mechanisms, such as the RAS/MAPK signaling pathway, also play crucial roles in the development and progression of TGCTs. Recent studies, such as those by Onorato et al., highlight the significance of these pathways, which interact with epigenetic mechanisms, potentially influencing the methylation status of key genes involved in tumor behavior [69]. Understanding these interactions could offer new insights into comprehensive therapeutic targets beyond epigenetic modifications alone.

Other studies found the hypermethylation of genes of interest, such as RFPL3, which codes for a testis-specific lncRNA and acts as a tumor suppressor in TGCT [64]. In the mentioned study, the expression profile of RFPL3S was able to differentiate between seminoma and non-seminoma as well as predict proliferation and metastasis and even immunotherapy response. When downregulated, RFPL3 was associated with poor prognosis, which could indicate *RFPL3* hypermethylation as a prognostic biomarker [64]. Another hypermethylated gene is *KCNC1*, whose downregulation was able to predict poor survival in seminoma patients and was suggested as a diagnostic biomarker as well as a therapeutic target after being identified through TCGA and confirmed seminoma cell lines [63]. In NSGT, high methylation levels of *BRCA1*, *RAD51C*, *PALB2*, *RAD54B*, and *SYCP3* genes were associated with their downregulation [65]. These genes are involved in DNA repair by homologous recombination. Thus, their methylation levels were suggested to be predictive of NSGT subtyping and staging as well as indicative of response to PARPi treatment. Similarly, the results were initially obtained on TCGA data and further confirmed in cell lines and, importantly, on paraffin-embedded samples from 150 patients [65].

Another pan-cancer TCGA study looked at the expression of the six DNases and their association with tumor stemness as a measure of cancer cells dedifferentiation [70]. The DNA methylation pattern was used for generating a DNA stemness score and, additionally, an RNA stemness score was based on mRNA expression. Among the cancer types included, TGCT stood out as having the strongest negative correlation between DNASE1 and RNA stemness, as well as DNASE1L1. Concerning methylation, however, DNASE1L3 was positively correlated the strongest with DNA stemness score in TGCT among all cancers [70]. Notably, in a different study, DNA stemness score was also positively correlated with the overexpression of a gene, namely *PDLC3*, and no correlation with overall survivor or progression-free survival was found [71]. In a study on the signal and transducer and activator of transcription (STAT) family, however, a negative correlation between DNA stemness score and *STAT5A* expression was found in TGCT, while this gene’s overexpression was strongly associated with poor overall survival [72].

In another pan-cancer analysis, the expression of the FAM72 family was generally upregulated in most tumors, including TGCT [73]. Interestingly, the member *FAM72A* was downregulated in TGCT as opposed to most cancers. This member’s expression seems to have been positively correlated with DNMTs expression and was further studied in relation with cancer stemness. It included methylation-based stemness scores, but no significant association was found in TGCT [73]. *FAM99A* gene, which codes for an lncRNA, had a lower expression in TGCT compared with normal tissue of the GTEx database; however, further investigations found a positive correlation with four methylation probe sites, and no prognostic value was considered for this cancer type, given the limitations of the study [62]. Interesting results were also obtained in a study on *SMARCA1*, a gene that encodes for a chromatin remodeling protein, which only evaluated its expression and DNA methylation due to limited data [74]. Despite low levels of expression, when looking at DNA methylation levels, the authors found two CpG sites in the promoter region that were negatively correlated with expression and another two CpG sites outside the promoter region with a positive association with expression [74].

The TCGA dataset was further used to develop an algorithm for identifying tissue of origin and tumor type in primary and metastasized cancer based on DNA methylation alterations [75]. The method developed by the authors, called HiTAIC, used a hierarchical model to identify 27 different cancers based on specific CpGs. In this hierarchy, TGCTs were classified in the first layer and showed a high precision, both in their test data set and the external validation data set. Furthermore, HiTAIC was able to accurately predict metastases to lung and lymph nodes, but only on a sample size of six patients [75].

Using data from TCGA, Gao et al. were able to develop a prognostic nomogram upon identification of a seven CpG sites-derived risk signature, which serves to predict progression-free survival (PFS), infiltration of immune cells, and chemotherapy sensitivity [66]. Their results indicated a total number of 1452 upregulated or downregulated genes between the high- and low-risk groups, which upon gene enrichment analysis showed association with immune-related biological processes or extracellular matrix tissue organization processes, respectively. Of particular importance, the high-risk group showed decreased sensitivity to conventional therapy with bleomycin and etoposide [66]. Although many differentially methylated sites between the without-progression and with-progression group were discovered, the seven selected sites were associated with good PFS when hypermethylated except for one hypermethylated CpG site, which predicted poor PFS. Some of the CpG sites were located near genes, namely *PPM1D*, *PANX1*, *ENDOD1*, *MAF*, and *MYH2*; others were near enhancers, and one was near a DNA-I-hypersensitive region [66].

Previously, an eight-gene panel was generated for stratifying patients with stage I TGCTs into low and high risk of relapse, but the study only included gene expression, with data corroborated between TCGA and GTEx [46]. The genes (*GPR174*, *TCTEX1D1*, *TMEM89*, *CST2*, *SRARP*, *GSC*, *PEKHS1*, *FLG2*) included in the risk score model may be further investigated to detect methylation status or mutations in order to further our understanding and support their use [46].

While most of these studies found several potential biomarkers with prognostic value in pan-cancer, the diagnostic value was evaluated extensively in a few recent studies. Of them, *SLC2A1* and *SMARCA1* had a high accuracy in predicting TGCT, which was calculated from the ROC (receiver operating characteristic) curves. Both had an AUC (area under the curve) higher than 0.9 (0.960 and 0.910, respectively) [59,74].

When identifying biomarkers with potential clinical application, it is necessary to confirm discoveries in patient samples, not only cell lines or animal models. For instance, a potential biomarker, HAVCR2 (hepatitis A virus cellular receptor 2) was investigated in a cohort of 15 patients with TGCT [76]. *HAVCR2* gene codes for a TIM (T-cell immunoglobulin and mucin domain-containing protein) family member and was found to be upregulated and positively correlated with immune infiltration and responsiveness to immunotherapy. To look at its methylation status, the authors integrated results from TCGA, which indicated hypomethylation in TGCT patients. Their study showed potential for the use of HAVCR2 as a diagnostic and prognostic marker for the 5-year survival rate. However, DNA methylation was not the only mechanism associated with its expression, as an increase in HAVCR2 copy number also led to upregulation. Furthermore, HAVCR2 expression levels were higher in seminoma patients compared with non-seminoma patients [76].

Unfortunately, however, confirmatory studies were rarely performed in patients for previously identified differentially methylated genes. Specifically, *MGMT* hypermethylation was strongly associated with non-seminomas and with poor prognosis in one study (also mentioned in the previous section) that looked at a gene panel that additionally identified *CALCA* methylation as a prognosis marker as well as a positive association between *MGMT* and *CALCA* methylation [31]. Markulin et al. could not replicate this result in their cohort but identified RASSF1A as a risk factor [29]. Soon after, both *MGMT* and *RASSF1A* were confirmed as having prognostic value and discriminatory power between subtypes, even in the case of mixed tumors, when combined with the methylation status of the additional genes *CRIPTO, HOXA9*, and *SCGB3A1* [77]. Intriguingly, *MGMT* and *RASSF1A* methylation was investigated in a cohort of 41 patients from India, of whom 24 were seminoma patients, 4 were non-seminoma patients, and 13 had mixed tumors [78]. When looking at *RASSF1*, 71%, 25%, and 46% of the corresponding groups showed methylation. In the case of *MGMT*, the results contradicted previous mentions, with none of the non-seminoma patients displaying methylation [78].

Moreover, the aforementioned genes were not amongst the top genes later identified through bioinformatics by Bo et al., whose study highlighted 8 differentially methylated genes of interest (*GAPDH, VEGFA, PTPRC, RIPK4, MMP9, CSF1R, KRAS, FN1*) among 604 hypomethylated and highly expressed genes and 147 hypermethylated and low expressed genes in TGCT [57]. On the other hand, some of these genes were included in a later in silico analysis performed on *MGMT, RASSF1A, PRSS21, CFC1, MAGEC2, OCT4, SOX17, SOX4, SALL4, NANOG*, and *KIT*. Most of them were hypomethylated in seminomas and hypermethylated in non-seminomas except for *SOX2* and *SOX17*, which showed no significant differences, and *SALL4*, which had an opposite pattern [79]. However, none of the above were amongst the hub genes identified in a study that integrated co-methylated and co-expressed genes in non-seminomas versus seminomas, with the hypomethylated and upregulated group including a series of 39 genes, while the hypermethylated and down-regulated group included only 2 genes, namely *ZNF438* and *FAM41C* [80]. Furthermore, one of the genes hypomethylated in non-seminomas *USP13* had been previously associated with hypermethylation in a cohort of embryonal carcinoma patients, possibly due to tumor heterogeneity [81].

While long-standing studies have focused on the hypermethylation of tumor suppressor genes as the most promising field for methylation-based biomarkers, here we present a growing body of work showcasing the potential of hypomethylation [13]. The use of liquid biopsies could focus on cfDNA methylation detection in blood for the diagnosis and prognosis of TGCTs, as indicated by recent advancements [37,79]. Comparing blood samples with tumor tissue obtained through orchiectomy could provide confirmation that the tumor is responsible for the modified parameters in blood. When looking at tissue samples, it is important to note that tissue adjacent to the tumor is usually used as a control, under the assumption that this tissue is normal. However, it is impossible to ascertain whether adjacent tissue is completely normal or whether some molecular modifications have taken place; thus, some caution must be considered when interpreting data. Additionally, the GTEx database makes use of tissues from deceased individuals [82].

## 4. DNA Methylation and Therapy

While some of the mentioned genes have been associated with response to immunotherapy, the current understanding of immune system activation in TGCT is limited. The most common type of immunotherapy involves the use of immune checkpoint inhibitors, which so far has led to disappointing results in this cancer type [83,84,85,86,87]. CAR-T cell therapy, however, has shown more promising results recently in a small cohort of patients and could provide a new avenue of treatment in patients whose only option so far is chemotherapy [88]. Future insights into immunological mechanisms and the involvement of methylation in their regulation may lead to general use of immunotherapy as well as targeted biomarkers [87].

Due to the nature of TGCT, it is important to look at the relationship between DNA methylation and the response to conventional therapy. While TGCTs typically respond well to cisplatin-based therapy, in some instances, cisplatin resistance occurs, as has been noted. It has been proposed that an epigenetic uncoupling is responsible for this instance, as it appears that when chemosensitivity and tumorigenicity are coupled, malignant TGCTs can either die through apoptosis or differentiate to benign teratoma that can be surgically removed [11]. When the uncoupling takes place, cisplatin-resistant cells develop and display tumorigenicity, which impedes treatment. Supposing that disturbed epigenetic mechanisms are responsible for this coupling, the use of epigenetic treatments could restore the default, highly treatable scenario. This model of resistance bears the name “rock and hard place”, deemed by Singh et al. [89]. While little evidence remains in this direction, some studies have tried to pinpoint the exact molecular changes that lead to resistance [89,90,91,92]. Spinella’s group looked at global DNA methylation levels in four TGCT-derived EC cell lines modified for cisplatin resistance through exposure–recovery experiments [91]. Their study showed global remodeling of DNA methylation in cisplatin-resistant cells, with novel hypermethylated/hypomethylated loci associated with chromatin reorganization and an increase in both CpG and non-CpG methylation. Notably, downregulation of tumor suppressor genes was observed as a possible consequence of DNA methylation at non-CpG sites [91].

Another study serving as a proof-of-concept was performed on a cohort of four patients, in which tumors were separated into individual components corresponding to yolk sac tumor, teratomas, embryonal carcinoma, and seminomas [92]. The patients had metastases that were also processed; thus, 12 total samples were obtained from formalin-fixed and paraffin-embedded tissues. When looking at matched primary tumors and corresponding metastases, as well as cisplatin-sensitive versus cisplatin-resistant tumors, the authors found that histological type was the major determinant of methylation, with different histological subtypes showing distinct DNA methylation patterns and limited clustering based on cisplatin resistance. Notably, an increase in overall methylation levels was observed in cisplatin-resistant embryonal carcinoma compared with the corresponding cisplatin-sensitive patient, which was also associated with poor outcome, thus being one of the few recent studies providing subtype-specific information. However, methylation patterns showed heterogeneity, even amongst histology pairs and patients. The results provided further basis for the use of demethylating agents in combination therapy in order to epigenetically prime these tumors, as was suggested by previous studies [83,92,93]. These studies built on previous work that argued for the use of DNA methylation inhibitors upon discovery of a relationship between hypermethylated promoters of particular genes and cisplatin resistance, which was the case of Martinelli’s results concerning *MGMT* and *CALCA* [31].

Additionally, a different rationale for using combination therapy with DNMTis comes from studies that identified abnormal expression patterns for DNMTs. Lobo et al. showed an upregulation of DNMTs, particularly of DNMT3B in a sixth of TGCT patients from TCGA database. Their expression pattern also showed discrimination power between subtypes and different stages. DNA demethylases were also investigated with comparable results. Mainly, DNMTs and TETs are negatively correlated, with lower expression of DNMTs and a higher expression of TET2 in seminomas, which supports the findings of a globally hypomethylated genome in this subtype. Conversely, DNMTs and TET2 have higher and lower expression, respectively, particularly in embryonal carcinomas and in advanced stages [94]. Similar results were previously published by other research groups, supporting the use of DNMTis [95,96,97,98].

5-azacytidine and 5-aza-2′deoxytidine, both nucleoside analogs, have received approval from the Food and Drug Administration (FDA) and the European Medicines Agency (EMA) for the treatment of hematological malignancies [94]. While their application in treating solid tumors has not yet gained official approval, pre-clinical research has shown that these drugs possess cytotoxic properties against such cancers. The effectiveness of 5-azacytidine is particularly noted in cases with elevated DNMT3B expression, suggesting its potential utility in managing TGCT, especially the highly aggressive non-seminomatous type, embryonal carcinoma, known for its high DNMT3B enzyme levels [99]. In preclinical studies, 5-aza-deoxycytidine was able to induce apoptosis of nullipotent and pluripotent EC cells, a process that is mediated by DNMT3B [99,100]. In cell lines, 5-aza was effective as a single agent in both cisplatin-sensitive and cisplatin-resistant cells. Moreover, it also managed to partially re-establish sensitivity in the resistant EC cells, which supports combination with cisplatin-based chemotherapy [101]. A summary of the clinical trials investigating DNMTi use in TGCT treatment is provided in Table 3.

The trials presented in Table 3 underscore the exploration of epigenetic alterations as a novel strategy to combat platinum resistance in GCTs, with a focus on utilizing DNA methyltransferase inhibitors to potentially reverse the resistance mechanisms. The use of SGI-110, in particular, represents a promising approach to addressing the challenges faced by patients with refractory GCTs, laying the groundwork for future clinical investigations in this area.

Agents designed to decrease methylation levels can mitigate the effects of hypermethylation, potentially reestablishing normal gene activity. Broadly, these agents fall into two categories: (a) nucleoside and (b) non-nucleoside inhibitors of DNA methylation. Table 4 concisely lists several examples from the two categories previously mentioned.

## 5. DNA Methylation and Fertility Implications

Testicular cancer poses a great threat to patients’ fertility, especially because its prevalence is particularly high in young men. The effects of therapeutic agents on DNA integrity have been studied in sperm samples, showing DNA damage even years after treatment [113,114]. Current therapeutic approaches for TC treatment are not specific to cancerous cells, affecting both tumors and normal cells. Furthermore, upon anticancer treatment, the male germline can also suffer epigenetic mutations, such as alterations of DNA methylation patterns, which are more subtle than genetic mutations and could be passed down [115]. Given the implied connection between TCs and epigenetic mechanisms, it would be of interest to look at the sperm epigenome post-treatment as its dysregulation could affect sperm quality and have a negative effect on fertilizing ability, or even on future offspring’s health [116]. However, few studies have examined this so far. Chan et al. have looked at global and locus-specific DNA methylation in semen samples provided by TGCT patients both prior and post-treatment [117]. As genetic reprogramming is a key event during germ cell and embryo development, the methylation status of several imprinted genes, including *H19, MEST, KCNQ1OT1*, and *SNRPN,* was analyzed in control samples, pre-treatment, and post-treatment samples. The results showed normal methylation levels in all groups; thus, imprinted genes were not affected by anti-cancer treatment. Regardless, the authors identified significant differences between control and TC patients concerning DNA methylation, with Illumina 450 K arrays showing distinct methylation signatures both prior to therapy and persisting for the entire period studied. Particularly, most affected CpG sites were hypomethylated compared with controls, and several genes’ promoters contained regions of differential methylation, of which *GDF2*’s was the largest. Moreover, methylation defects increased after treatment and were subsequently resolved, with some persisting up to 2 years. While the number of patients included in the study was limited, as there were only seven patients with NSGCT and eight control volunteers, and while not all patients provided samples throughout the entire 3.5 years of follow up after treatment, these results show that sperm DNA methylation defects can persist in NSGCT patients, which is of particular concern for their off-spring as it could lead to birth defects, with one possible explanation being the survival of spermatogonial germ cells with abnormally methylated DNA post-treatment [117].

A longitudinal case study of a 30-year-old patient showed similar results, in which 17 of the 74 differentially methylated regions identified in sperm samples prior to treatment remained altered 2 years after treatment but no imprinted genes were affected [118]. Interestingly, among them was a histone methyltransferase gene, *KMT2C*, which is essential during early embryogenesis. Additional differentially methylated genes associated with embryo development included *SCAP, GBX2, PTK2* (hypermethylated) and *BCR*, *SH2D4B*, *SATB2*, *SLC39A3*, and *HPN* (hypomethylated). While most of the alterations do not persist years after treatment, defects related to embryo-essential genes are of great concern. Despite limited evidence, further studies may be able to fully assess the sperm of cancer survivors [118].

Given the challenges of obtaining healthy spermatozoa after therapy, another direction of study focuses on fertility preservation by the cryoconservation of sperm obtained prior to treatment initiation. Spermatogonial stem cell transplantation (SSCT) is a viable option as it preserves stem cells obtained through testicular biopsy that can be propagated in vitro at a later time and transplanted into the healthy testis. This has been proposed as a solution for childhood cancer survivors that want to preserve their fertility until they reach adulthood. Due to the environmental stress of cell culturing, however, there are concerns for epigenetic modifications, such as altering the sperm methylome and passing alterations to their children [119,120]. In addition to genetic and epigenetic studies, animal models have provided valuable insights into TGCT pathogenesis. Although studies such as those by Guida E. et al. primarily focus on signaling pathways like MAPK, they contribute to the broader understanding of tumor biology [121]. Such models are crucial for testing the implications of DNA methylation findings in a controlled, experimental environment and could potentially reveal interactions between methylation patterns and other cellular pathways. Serrano et al. looked for such effects in mice and found one gene with an altered methylation pattern, *Tal2*, which was hypomethylated in F1 offsprings sperm and had a higher expression in F1 offspring ovaries [122]. Nevertheless, no major differences were found in the DNA methylation pattern of sperm obtained from F1 and F2 generations, with only less than 0.5% of all differences in hyper- or hypomethylation patterns being statistically significant. When looking at *Tal2*, the higher expression was resolved in the F2 generation, but concern remains for the F1 generation as some studies have associated *Tal2* with ovarian cancer, though usually due to decreased expression [122,123].

A previous study into the potential use of SSCT used spare testicular tissue fragments to generate cell cultures and compared them with primary seminoma tumors, with respect to their DNA methylation status [124]. The reported findings included the well-known hypomethylated profile of the seminoma tumors, which was not detected in the cultured cells. When looking at specific genes’ methylation profiles, no differential methylation occurred in the case of *PRSS21*, *HIC1*, or the previously mentioned *SCGB3A1*, while *KIT* was hypomethylated in all groups. Other genes displayed differences, because *OCT4/POU5F1*, a pluripotency marker, was hypomethylated in seminomas but not in uncultured or cultured primary testicular cells, while *DPPA3* and *XIST* were hypermethylated only in long-term cultured cells. The previously mentioned *MGMT* gene was also analyzed, and it displayed variable levels, even within the same groups. While the absence of some epigenetic modifications that are considered cancer signatures is reassuring, further studies should assess the safety of using these cells [124].

Besides alterations associated with cell culturing, the methylome of future offspring could be altered by the techniques used in in vitro fertilization. Intra-cytoplasmatic sperm injection (ICSI) is an invasive technique that involves direct injection of a selected spermatozoa into the cytoplasm of a mature oocyte. Oblette et al. looked at the effects of using in vitro-generated spermatozoa in mice and found altered DNA methylation levels, but no difference in certain histone modification levels, when compared with embryos derived from in vivo-produced spermatozoa [125]. While their study involved the use of ICSI in both groups, it would be of interest to look for alterations that are possibly caused by ICSI compared with a control group.

Overall, the results provided hope for the implementation of SSCT in humans in the future. Notwithstanding, if we consider both cancer itself and its treatment affecting male germ cells, then by the time TC patients freeze their sperm, it is already too late for some epigenetic changes that may have taken place. As such, restoring normal DNA methylation pattern in sperm cells would be a better solution, but it is currently unobtainable. However, fertility treatments involve processing sperm samples in order to select the best spermatozoa, which could provide an avenue for the development of an epigenetic-based selection method and thus lower the chances of TC survivors passing on defects to their children.

## 6. Future Perspectives

Future studies will increasingly focus on the comprehensive epigenetic profiling of TGCTs, utilizing cutting-edge single-cell epigenomics to dissect tumor heterogeneity and pinpoint epigenetic signatures critical for tumor development and progression. Understanding the mechanisms of epigenetic plasticity that contribute to treatment resistance is paramount. This knowledge will guide the development of targeted therapies aimed at specific epigenetic alterations, offering a promising avenue to overcome resistance to conventional treatments like cisplatin. Moreover, the emergence of targeted epigenetic interventions, including DNMTi and CRISPR-based epigenome editing tools, heralds a new era of precision medicine in TGCT therapy. These approaches hold the potential to not only improve treatment outcomes but also pave the way for combination therapies that target multiple tumorigenic pathways simultaneously, thereby enhancing efficacy and preventing resistance.

The advancement of liquid biopsy technologies and integrative omics analyses represents an important step forward in the battle against TGCTs. By harnessing the power of circulating tumor DNA (ctDNA) methylation patterns, liquid biopsies offer a non-invasive method for the early detection, ongoing monitoring, and prognosis of TGCTs, potentially revolutionizing patient management and improving outcomes. Furthermore, the employment of integrative analyses that merge genomic, transcriptomic, and epigenomic data promises to provide a comprehensive understanding of TGCT biology. This holistic approach not only opens new ways for the discovery of novel biomarkers but also enables the identification of some new therapeutic targets, thereby enhancing the precision and effectiveness of TGCT treatments. Together, these innovative strategies underscore the importance of a multi-faceted approach in improving diagnosis, treatment, and prognosis for TGCT patients, highlighting a future where personalized medicine becomes a reality.

In conclusion, the future of TGCT research and treatment is poised at the intersection of epigenetics and personalized medicine, promising improved outcomes for patients through innovation in diagnosis, treatment, and prevention strategies.

## 7. Conclusions

With the growing body of evidence on the subject, it has become increasingly obvious that DNA methylation has multiple ramifications into the understanding of TGCT as its involvement is ever-present, from the events leading to tumor initiation to progression and resistance to therapy. As such, current research is honing in on the potential use of DNA methylation in biomarker development and anti-cancer treatment. Even post-treatment, patients may benefit from the extensive research into DNA methylation through its use in advancements of fertility management.

The biggest limitation on TGCT research advancement seems to be its low incidence, as researchers struggle to obtain significant results with a small number of patients’ samples at their disposal. Most cohorts have a low number of subjects, which makes it hard for any discovery to be validated, coupled with the difficulty of publishing results obtained on few subjects or even one single patient. As few clinical trials have been initiated, TGCT patients do not have many options at their disposal when it comes to tests and treatments compared with other, more prevalent cancers, e.g., prostate cancer. Although the number of TGCT patients is on a rising trend globally, it is oftentimes difficult to enroll patients in research studies, particularly when multiple follow-up visits are needed. Providing valid samples may be challenging when treatment has already started, as is the case of semen samples due to therapy-induced azoospermia. Moreover, there are multiple subtypes of TGCT with distinct characteristics, including their epigenome, which makes it even more difficult to find an appropriate cohort. Also, many patients display mixed tumors, which makes interpreting results extremely challenging.

Given the limited number of patients, researchers have turned their attention to databases such as TCGA and GTEx for data they can use, followed by validating their results in cell lines, animal models, and small cohorts. This has led to an increase in the number of potential applications, as data are generated at a faster pace than ever before. Prudence must be applied when using this method, however, as current databases also have limitations when it comes to TGCT patients. Importantly, there is a need for standardization when investigating DNA methylation, as differing results between study groups may be due to a lack of consensus in sites used for methylation status evaluation.

Overall, DNA methylation research has not reached its full potential in relation to TGCT association. Therefore, there is still hope for a better understanding of the molecular characteristics of TGCTs and their further exploitation through the development of non-invasive biomarkers, new therapeutical strategies, and improved fertility management, thus leading to a superior standard for patient care.

## Figures and Tables

**Table 1:** Concise classification of the most prevalent types of testicular germ cell tumors.

Category	Family	Type
GCNIS-derived (known as type II)	Non-invasive	GCNIS, gonadoblastoma, etc.
	Germinoma	Seminoma
	Non-seminomatous	Embryonal carcinoma
		Yolk sac tumor, postpubertal
		Choriocarcinoma
		Teratoma, postpubertal
	Mixed	Mixed germ cell tumors
Unrelated to GCNIS		Spermatocytic tumor (known as type III)
		Teratoma, prepubertal (known as type I)
		Yolk sac tumor, prepubertal (known as type I)

**Table 2:** Most recently investigated genes’ methylation status and their roles.

Target	Potential Role in Relation to Cancer	Expression Level	Methylation Status	References
AKT3	Tumor progression Immune infiltration	Upregulated	Hypomethylated	[60]
CSF3R	Tumor immune infiltration			[56]
NTRK3	Tumor initiation and progression			[54]
SLC2A1	Cancer growth Immune infiltration Overall high diagnostic and prognostic value			[59]
SMARCA1	Tumor progression Immune infiltration High diagnostic value			[62]
KCNC1	Diagnostic, subtyping and prognostic value, therapeutic target	Downregulated	Hypermethylated	[63]
RFPL3	Diagnostic, subtyping and prognostic value			[64]
BRCA1	DNA repair by homologous recombination Subtyping, staging, therapy response			[65]
RAD51C				
PALB2				
RAD58B				
SYCP3				
PPMD1	Tumor suppressor gene	-		[66]
PANX1	Cancer growth			
ENDOD1	Cell death			
MAF	Cell proliferation			
MYH2	DNA repair			

**Table 3:** Clinical trials with epidrugs targeting DNMTs.

Target	Drug	Combination	Tumor Type	Status	Trial Number	References
DNMT	5-azacytidine	Single-agent activity	Refractory GCT patient	Completed	-	[102]
	5-azacytidine	-	Advanced GCT	Completed	-	[103]
	SGI- 110 (Guadecitabine)	+Cisplatin	Refractory GCT Patients	Phase II	NCT02429466	[104]
	SGI-110	+Carboplatin	Platinum refractory ovarian cancer Refractory GCT cell lines to platinum	Phase II	NCT01696032	[102]
	5-aza-4′-Thio-2′-Deoxycytidine (Aza-TdC)	-	Neoplasms, solid tumors	Recruiting	NCT03366116	[105]
PARP +DNMT	Gemcitabine	Veliparib and carboplatin		Completed	-	[85]

**Table 4:** Overview of the selective hypomethylating agent and their current research status.

Modifier Type	Drug Example	Description	Current Research Phase	References
DNA methylation inhibitors (bind directly to enzymes)	Azacitidine analog	An agent that directly binds to DNA methyltransferases with recognized therapeutic efficacy but is limited by its considerable toxicity and reduced half-life.	Authorized for use	[106]
DNA methylation inhibitors (integration into DNA)	Decitabine	A modified nucleoside that merges into DNA, enhancing selectivity due to its unique incorporation, extending the half-life over its counterparts.	Authorized for use	[107]
DNA methylation inhibitors (stabilization enhancement)	SG110	A compound that modulates the stability of the DNA methyltransferase inhibitor, leading to diminished drug resistance and prolonged action.	Clinical trials underway	[108]
DNA methylation modulators (nucleoside transport alteration)	CP4200	A derivative aimed at augmenting intracellular transport efficiency, albeit at the possible expense of binding precision.	Experimental stages	[109]
Enzyme competitive inhibitors	Cladribine	This substance hinders a key enzyme, potentially heightening the concentration of a critical methyl group donor, which could impede DNA methyltransferases.	Authorized for use	[110]
DNA methylation inhibitors (active site binding)	Procainamide	Binds with high specificity to DNA methyltransferase 1, thus potentially halting DNA methylation processes.	Authorized for use	[111]
Natural epigenetic compounds	Genistein	A natural isoflavone that may reduce DNA methyltransferase activity, with uncertain impacts on DNA methylation patterns.	Advanced research stages	[112]
